# Birthplace as a capital: migratory flow, labor opportunities, and social reproduction in Brazilian men elite futsal players' careers

**DOI:** 10.3389/fsoc.2025.1487326

**Published:** 2025-06-04

**Authors:** Iuri Salim de Souza, Murilo dos Reis Morbi, Illgner Veber Garcia Alves, Christiano Streb Ricci, Renato Francisco Rodrigues Marques

**Affiliations:** ^1^University of São Paulo, Ribeirão Preto, Brazil; ^2^Marista School, Ribeirão Preto, Brazil; ^3^University of Ribeirão Preto, Ribeirão Preto, Brazil

**Keywords:** domestic move, labor mobility, sport career, Pierre Bourdieu, reflexive sociology, social inequality, social reproduction

## Abstract

**Introduction:**

This study investigated the association between social inequality and the sport labor career development opportunities in Brazil. We analyzed the interrelation of Brazilian men's elite futsal players' birthplaces, the clubs' locations, and the intra-national (domestic) migratory flow among regions and states within this country. Brazil is a Global South country with a high level of social inequality where futsal is a very popular sport, with one of the most relevant men's national leagues worldwide—the *Liga Nacional de Futsal* (LNF). This league commonly counts on around 20 clubs, all located in the South or Southeast regions of Brazil, which are socioeconomically wealthier and with a higher Human Development Index (HDI). The aims of this study were: (a) to analyze how men professional futsal players' migratory flow occurs in Brazil, considering and relating the athletes' birthplace and the clubs' location regions; (b) to investigate players' working period in the same club and the tendencies of instability/stability in job positions; (c) to analyze relations between the socioeconomic inequality in different Brazilian regions and the athletes' migratory flow.

**Methods:**

With a quantitative research approach, we analyzed the birthplaces and migratory flow of LNF players between 2013 and 2022. Data discussion was based on Bourdieusian Reflexive Sociology. Results showed that: LNF clubs are located in Brazilian socioeconomic richest regions, with most players born there; a minority of athletes remained working in the same club for 3 years or more.

**Discussion and conclusion:**

We concluded that Brazilian men's elite futsal context reproduces socioeconomic inequalities through a very regionalized athletes' migratory flow, and provides unstable labor conditions to players, who remain for short periods joining the same club.

## 1 Introduction

### 1.1 Context and hypotheses

This study investigated the association between social inequality and the sport labor career development opportunities in Brazil. We analyzed the interrelation of Brazilian men elite futsal players' birthplaces, the clubs' locations, and the intranational migratory flow among regions and states within this country.

Brazil is a Global South country with a high level of social inequality (Graeff et al., 2019; Eguiluz et al., 2022), being the 5th largest and the 7th most populous nation in the world (Worldometer, 2023). This country has a huge cultural diversity across its territory, influencing practices/manifestations in sport, art, food, clothing, leisure, education, marketing, collective identity, and other social dimensions (Marques et al., 2016; Graeff, 2020).

**Figure 1:**
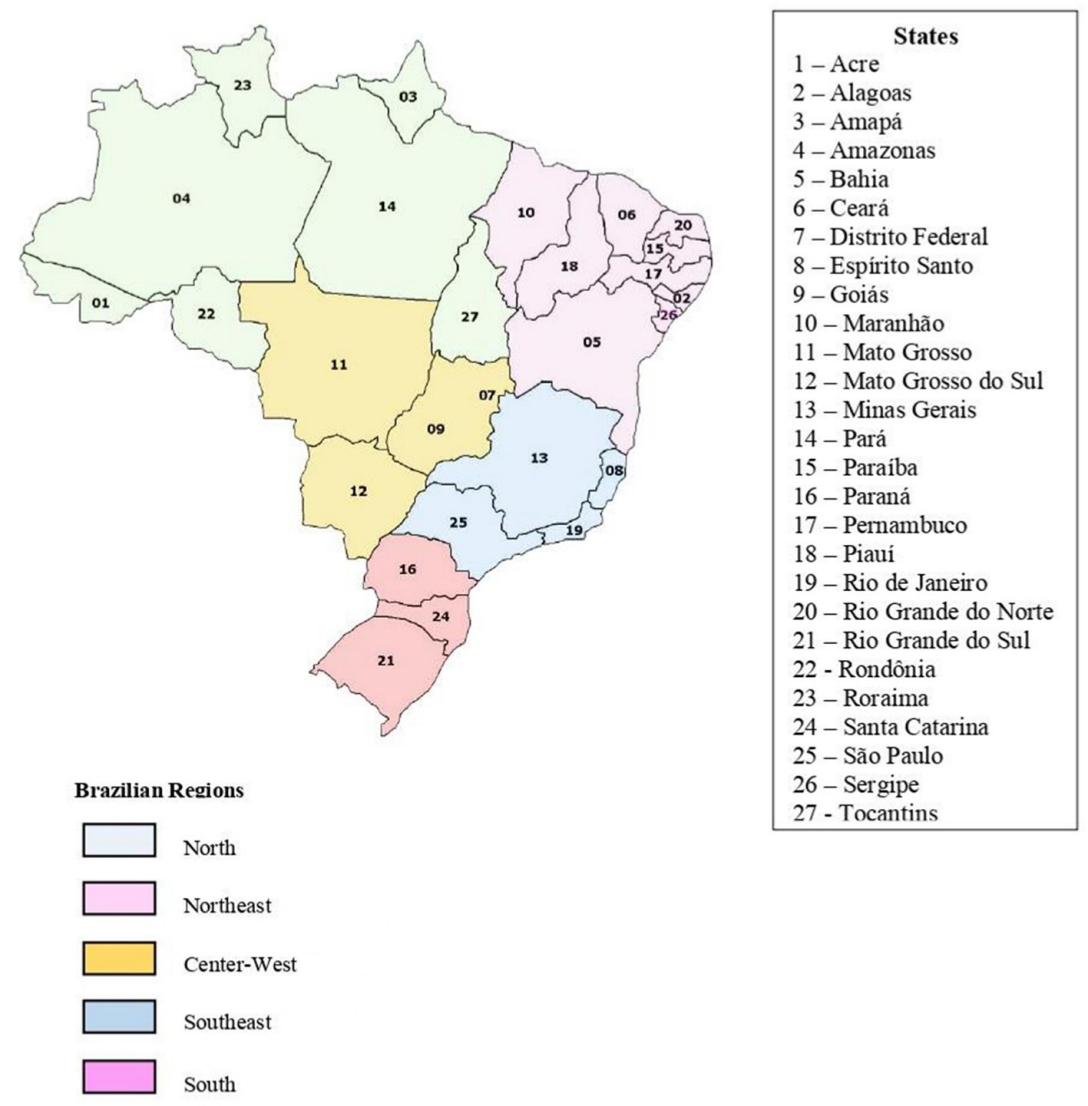
Brazilian federal regions and states. Source: Adapted from Wikimedia commons—Link: https://commons.wikimedia.org/wiki/File:Mapa_do_Brasil_por_regi%C3%B5es.PNG.

Brazil has a governmental organization divided into 26 states and a federal district, distributed among five regions (Figure 1)—North, Northeast, Centre-West, South, and Southeast, the latter being the most populous, socioeconomic richest, and presenting the highest Human Development Index (HDI) (Atlas Brasil, 2022). Converging to this, the most prominent and wealthier sport clubs are located in the Southeast region, followed by the South region, with both also concentrating the most of the major sport leagues, tournaments, and federations (Almeida and Rubio, 2021; Marques, 2024).

Futsal is a very popular sport in Brazil, with ~10 million practitioners (Santos et al., 2019), being the most practiced by children and adolescents in schools (Voser and Giusti, 2015). Furthermore, Brazil is the winner of the highest number of futsal world championships in both men's and women's scenarios (Mascarin et al., 2019).

Futsal in Brazil involves a complex sport system based on leagues, federations, and associations, with clubs across the country promoting senior (amateur or professional) and youth (under-7 to under-20) teams, with several players migrating from family homes since adolescence (Marques et al., 2022). In this country, investment in a professional futsal career can offer prestige and opportunities for social mobility to young and adult athletes, despite a scenario of many instabilities and informalities regarding the labor agreements/contracts between clubs and athletes (Marques et al., 2021).

There are several national senior-level men's futsal tournaments in Brazil, among regional, state, and micro-regional leagues, in all states of the country. Brazil has one of the most important men's national leagues worldwide, the *Liga Nacional de Futsal* (National Futsal League—LNF), which counted 24 clubs and around 400 players in the 2024 edition (LNF, 2024). An important dimension to consider is that most LNF clubs are located in the South and Southeast regions of the country, which can be regarded as an example of financial wealth concentration and social inequality within the national sport system.

The LNF has as its partner, between 2014 and 2023 editions, the Brazilian Futsal Confederation (CBFS), which nowadays has legal and economic struggles with the LNF. This scenario resulted in the creation of the Brazilian Futsal Championship, an alternative national competition under the control of the CBFS. This competition started in 2024 with 20 clubs in its first edition—8 from the Northeast region, 3 from the Centre-west, 2 from the North, 3 from the Southeast, and 4 from the South. According to the president of the CBFS, the goal of the new tournament is not to make it elitist or restrict it only to the Southern regions. According to this, the CBFS recruited futsal clubs from some of the most important clubs in the men's Brazilian professional football league. These clubs were part of the LNF in its early days, but lost their places to clubs located in the country cities in the Southeast and South regions (Vecchioli, 2023). However, the LNF, as the major futsal league in the country, is present in the wealthier regions (South and Southeast), establishing a strong control over the athletes' market. As the LNF dominates the field, this position attracts more sponsors and income through broadcast rights on television and streaming channels, creating commercial barriers to the CBFS initiatives.

Professional athletes tend to migrate to where there are better labor opportunities (Roderick, 2012; Agergaard et al., 2023). In the case of LNF, they are concentrated in the Southern regions. Considering this, our first hypothesis for this study is that social inequality is a relevant factor that influences the athletes' migratory flow and the development of their careers. This social condition makes athletes' mobility harder for those born in regions without LNF clubs—North, Northeast, and Centre-West. The second hypothesis was that Brazilian men's elite futsal context could symbolize a mechanism of social reproduction. It would happen because the players born in the same regions where the wealthier clubs are located could have privileges to access job opportunities in the LNF, compared to those from other places.

To test these hypotheses, we used a quantitative method based on the following data: the birthplaces of Brazilian men futsal players who joined the LNF; the clubs' locations; the segmented men's Brazilian population birthplaces distributed by the country's regions; the migratory flow of Brazilian men elite futsal players among regions and states; the index of permanency of players in the same club (job stability). This methodological approach is coherent with the study's topic and hypothesis, providing results and answers related to the players' migratory flow and participation in LNF.

### 1.2 Rationale and aims

Professional sport is a context that involves high competition among athletes, not only related to performance or results, but also to labor career development (Elliott, 2016; Rojo and Starepravo, 2025). In the context of the Global South countries, several athletes struggle to access job positions that commonly present precarious circumstances, based on unstable, short, and informal job agreements (Besnier et al., 2018). One of the consequences of this phenomenon is the athletes' migration, as part of a search for labor opportunities that offer profitable economic gains and better working conditions (Marques and Marchi Jr., 2021). However, in several of the athletes' experiences, these goals are not achieved because of a certain precariousness in labor relations, which imposes a condition of vulnerability on these people (Roderick, 2006; Besnier et al., 2018).

Under the influence of neoliberalism in contemporary society, sport has become a representation of hope for social and economic individuals' legitimation. In this sense, athletes are expected to be flexible agents, capable of mobilizing themselves to accumulate the necessary capital to be part of a professional sport context (Besnier et al., 2018). However, to migrate, the athletes depend not only on their will but also on opportunities for a process with minimal or adequate conditions to be socialized and to work in another location. In other words, they must access capitals as resources for mobility (Marques et al., 2022). In a scenario where athletes are vulnerable, it is not always possible to migrate and/or continue developing their sports career (Agergaard and Ungruhe, 2016).

Considering that labor migration and job opportunities can be related to social inequality in South American countries (Takenaka and Pren, 2010; Robins, 2019) to analyze how the intra-national migratory flow of Brazilian men's professional futsal players is a way to understand the offer of possibilities for athletes' sport career development and their insertion in the elite sport labor market. This kind of investigation can reveal mechanisms for the changing or the reproduction of social inequality, through the analysis of the Brazilian men futsal players' careers and the possibilities of joining the major league in the country.

The high worldwide interest in new sport talents (Poli et al., 2018), including the Global South context, demands a better understanding of the migratory flow of athletes, both in transnational and intranational contexts (Carter, 2016). In the case of football and futsal, Brazil is a prominent place, being the origin of several elite-level players who move around the globe to work (Leeds and Leeds, 2009; Mascarin et al., 2019). A better understanding of sports labor conditions and the migratory flow of Brazilian athletes is a relevant topic to be analyzed, not only related to regional/national impact, but also to an international dimension (Darby et al., 2007; Carter, 2016; Spaaij et al., 2019).

To reflect on this social scenario, the aims of this study were: (a) to analyze how men professional futsal players' migratory flow occurs in Brazil, considering and relating the athletes' birthplace and the clubs' location regions; (b) to investigate players' working period in the same club and the tendencies of instability/stability in job positions; (c) to analyze relations between the socioeconomic inequality in different Brazilian regions and the athletes' migratory flow.

Within this context of social inequality among Brazilian regions, a balanced number of futsal athletes' mobility from their different origin places to where LNF clubs are located could be interpreted as a scenario of more equalitarian opportunities for developing a sport career. On the other hand, a large concentration of players born in the same regions where clubs are located (the wealthier ones in the country) could be understood as a context of social reproduction, where the opportunities to accumulate capitals (through career development) are more accessible to those players that have previous resources (as access to major clubs in their birthplaces). The scientific relevance of understanding this context can be justified as a way to offer theoretical subsidies for sport management and public policy stakeholders. This body of knowledge can provide insights for actions against social inequality within the Brazilian sport field and support other places' sports context analysis and interventions.

Results from this study, developed in a Global South country, are relevant because they present a context of social inequality not so well explored by the literature on Sport Migration. They can also support both the implementation of sport policies, considering the migratory flow of athletes in Brazil, and provide reflections that can influence other countries' landscape analyses on social inequality and the offer of opportunities for sport labor and career development. Furthermore, Brazil is a sending place of futsal migrant athletes to several countries in Europe, Asia, and North America, being mainly the origin and not the destination of them (Poli, 2010; Gavira et al., 2013; Poli et al., 2017; Poli and Ravenel, 2018). However, the majority of sport migration studies focused on data from receiving places and socioeconomically richer countries, mainly from the Global North (Gavira et al., 2013).

Despite futsal's social relevance in Brazil, few studies on men futsal players' labor migration are available in the literature. A small group of articles presents issues and challenges mainly faced by Brazilian men players during their experiences of transnational migration (Dimeo and Ribeiro, 2009; Tedesco, 2014; Marques and Marchi Jr., 2021; Oliveira Filho, 2022). Others analyzed the context of the intra-national migration of Brazilian National team players during youth (Marques et al., 2022), and in the context of adult athletes' labor issues (Marques and Marchi Jr., 2021). Within this scenario, Marques and Marchi Jr. (2021) argued that over the years 2013 and 2019, 11% and 13% of Brazilian men elite futsal players remained working at the same club/city for at least 3 years. These data show a high job instability landscape and a constant athletes' migratory flow. It is important to consider that each LNF club was located in a specific city, which means that every player's club change also meant a move to another town.

Considering all these contributions, the mentioned studies did not analyze the Brazilian LNF players' birthplace as an indicator of the migrant athletes' origin location to invest in a futsal career. This information is relevant because it can show if athletes from different places are receiving equitable opportunities to develop a professional career (Bruner et al., 2011). In the case of Brazilian men's futsal, this knowledge is also useful to provide a better understanding of where these players are moving to join LNF clubs, and a possible necessity of intervention to provide good labor opportunities to athletes from different regions of the country.

Considering that the sociological approach is very suitable for understanding the migratory flow of athletes (Elliott and Maguire, 2008; Carter, 2013; Agergaard et al., 2023), to analyze Brazilian futsal players' migration experiences, we based our study on some categories from Pierre Bourdieu's Reflexive Sociology (Bourdieu and Wacquant, 1992; Bourdieu, 1998). Furthermore, we considered his contributions and reflections on migration as a social phenomenon, and a consequence of the social precariousness experienced in several social conditions/scenarios (Bourdieu and Wacquant, 2000; Bourdieu, 2004; Bourdieu and Sayad, 2004).

Within this context, the choice of Pierre Bourdieu's Reflexive Sociology as a theoretical framework for this study is justified because of this author's approach to social inequality within the neoliberal society, and his problematization of unequal access to capital as a process that influences agents' dispositions for action and the possibilities of social participation. According to this, we will mainly sustain our analysis on the Bourdieusian concepts of field, capital, and social reproduction.

## 2 A Bourdieusian approach to migration studies

Pierre Bourdieu's Reflexive Sociology has some important topics for analysis of social inequality, the struggles for capitals, and the ways the hidden mechanisms of the reproduction of social stratification operate (Bourdieu, 1984, 1993). Adding to this, he also provided a consistent analysis of social and labor relations within the neoliberal system (Bourdieu, 2001).

Bourdieu proposed that individuals interact influenced by social rules and stratified structures through several fields, partially autonomous social spaces, with their own rules and history. In the fields, agents and groups are differently positioned, shaping social structures that are configured by the unequal distribution of resources, which creates different social positions (Bourdieu, 1993). Sport is an example of a field (Bourdieu, 1978, 1984, 1988, 1993), as well as its subfields, which, despite having some particularities, respect the major common structure. In this case, futsal can be considered a subfield of sport (Marques et al., 2021).

The social positions occupied by agents and groups within the fields, as their dispositions for action, depend on their access to resources that, when legitimated, can provide power and social recognition (Bourdieu and Wacquant, 1992). These resources are called capitals. Bourdieu proposed four types of capitals: (a) Economic capital, which relates to the possession of money and financial resources; (b) Social capital, which derives from agents' networks and interpersonal relationships. It is the agent's recognition by other agents or groups that have social legitimation; (c) Cultural capital, which relates to embodied knowledge and the possession of goods with cultural value, such as academic certification, books, and pieces of art, among others; and (d) Symbolic capital, which is the forms of capital that in particular contexts are highly valued, according to the rules and social norms of the different fields. The agents can accumulate capitals either through accumulated labor (Bourdieu, 1986) or as an inheritance, transmitted within families (Bourdieu and Passeron, 2014). Capitals are linked among themselves through conversions, when a type of capital can be converted, or transformed, into another. An example is the symbolic power provided by an academic certification (Cultural capital) that produces an increase in the salary of the individual (Economic capital). A similar example can be seen in the sport field. Where the athletic skills or the sport reputation (Symbolic capital) of the agent can be converted into the opportunity to join a better-ranked club, providing higher salaries and other possibilities for financial gains (Economic capital).

According to Bourdieu, the more individuals are well-positioned in the social structure of a field, the greater their chance to accumulate more capitals and power. It also affects those agents who have difficulties accumulating capitals, making it even harder to get their social mobility, because as far they are from these resources, the harder to access new forms of it (Bourdieu, 1977). This process can be called social reproduction. It imposes a tendency to maintain the social structure of the field (Bourdieu, 1973).

The possession of capitals can provide the agent with the power of symbolic violence, in other words, a privileged social position in interpersonal relations, and to decide on the ways of capitals distribution in the field (Bourdieu, 1993). This symbolic power is a product of the agent's social recognition in the field, through the influence of its capitals, being legitimated and reproduced by *doxa*, the social order accepted by agents (Bourdieu, 1998). To those social agents, who accumulate the capitals and the power to define the criteria for their distribution, there is commonly an interest in the reproduction of the social structure. It can be called an orthodox way of agency. On the contrary, those agents that have less access to capitals tend to act through strategies of subversion and social change, configuring a heterodox agency (Bourdieu, 1993).

Within this context, the agents' dispositions for action can be called habitus (Bourdieu, 1998), “…a system of dispositions acquired by implicit or explicit learning which functions as a system of generative schemes” (Bourdieu, 1993, p. 76). Habitus simultaneously works as a structure to agents' perception and acting in the fields, structured by their social position and accumulated capitals, but also influences the functioning of the social structure (Bourdieu, 1984). It works as “…a system of durable, transposable dispositions, structured structures predisposed to function as structuring structures” (Bourdieu, 1977, p. 72). In each new agent's social position in the fields, its habitus undergoes transformations (Bourdieu and Wacquant, 1992).

Considering this sociological approach, migration can be considered a strategy to maintain or improve the migrant's social position in a specific field (Bourdieu and Wacquant, 2000). However, while this expectation is attractive, the migrant becomes a displaced person during the moving process (Sayad, 2004, 2008). Moving involves an inherent relationship between groups' backgrounds and migration practices (Rye, 2011). Because of this, it is a process of production and reproduction of symbolic power and social structures through actions and practices, in different communities and networks (O'Reilly, 2013, p. 3).

Migration is a socio-geographic phenomenon that can be analyzed from the sending and/or receiving places' point of view (Bourdieu, 2004). Considering a Bourdieusian approach to migration studies, this analysis involves understanding why and where people migrate, and how capitals influence the migrants' agency (Erel, 2010).

## 3 Method

This is a quantitative approach study, based on official data from LNF related to: the birthplaces of Brazilian men futsal players who joined the LNF; the clubs' locations; the men Brazilian population birthplaces distributed by the country's regions; the migratory flow of Brazilian men elite futsal players among regions and states; the index of permanency of players in the same club (job stability).

We analyzed the scenarios of four different LNF editions. We intended to compare the distribution of players according to their birthplaces and clubs, as a way to analyze some possible changes over 9 years and understand the scenario of migratory flow in the Brazilian men's elite futsal context. We also compared the distribution of players' birthplaces with the Brazilian percentage of the segmented men's population of the same age to analyze whether the players' sample is similar to what is widely observed in the country. We performed this procedure to investigate whether this study's results are related to the context of elite men's futsal as a particular scenario or whether they are reproduced in the general Brazilian population.

### 3.1 Sample

The population of this study was composed of Brazilian men futsal elite athletes who participated in at least one of the LNF 2013, 2016, 2019, and 2022 editions (CBFS, 2013; LNF, 2016, 2019, 2022). The choice for a three-year interval between seasons was based on the results from Marques and Marchi Jr. (2021) who indicated this period as the expected time of working in the same club by Brazilian men elite futsal players. The total data corpus of the four studied editions of LNF is 1,628 players.

During data collection related to the players' birthplace and the city where the clubs are located, some information was lost due to imprecision and/or data replication. Considering only the reliable data, the final sample of this study was composed of 1,343 Brazilian LNF players (foreign players did not participate in the LNF studied editions), being distributed as follows: LNF 2013—376 players; LNF 2016—223 players; LNF 2019—343 players; LNF 2022—401 players. The necessary number of players for a representative sample with a 95% confidence level was 311 athletes, with our sample being larger than this.

### 3.2 Data collection

The athletes' birthplace data were collected on the official websites of LNF 2013, LNF 2016, LNF 2019, and LNF 2022 (CBFS, 2013; LNF, 2016, 2019, 2022). Data related to the birthplaces of the Brazilian men between 0 and 14 years old population (segmented population) were obtained from two different websites that provided official data from the Brazilian Demographic Census: *Atlas do Desenvolvimento Humano no Brasil de 1991* (Atlas of Human Development in Brazil of 1991) (Atlas Brasil, 2022), and *Censo Demográfico no Brasil de 2000* (Demographic Census in Brazil of 2000) (IBGE, 2010). The first one was used to compare LNF 2013 and LNF 2016 athletes' birthplace distribution by regions with the men's Brazilian population between 0 and 14 years old in 1991. The second one was used to compare LNF 2019 and LNF 2022 athletes' birthplaces with the men's Brazilian population between 0 and 14 years old in 2000. This division was made to approximate the social context and demographic distribution in Brazil when athletes were born and also during their youth, considering that this study sample players' average age of the LNF 2013 and LNF 2016 was 25 years old, and the LNF 2019 and LNF 2022 was 26 years old.

To compare how many players worked at the same club during the interval time of three years, and analyze the migratory flow of athletes within this scenario, we first present the data from Marques and Marchi Jr. (2021) related to the analysis involving the LNF 2013, LNF 2016, and LNF 2019 (see Tables 1, 2). After that, we collected data from LNF 2022, providing a comparison with the LNF 2019, presented in the following results section.

**Table 1:** Players' working period in the same clubs between LNF 2013 and LNF 2016.

**Clubs of LNF 2013**	**Players in LNF 2013**	**Players that worked at the same club in both LNF 2013 and LNF 2016**	**Percentage of players that worked at the same club in both LNF 2013 and 2016**
A	11	1	9.1%
B	22	4	18.2%
C	16	2	12.5%
D	18	2	11.1%
E	23	8	34.8%
F	15	2	13.3%
G	27	1	3.7%
H	22	0	0.0%
I	19	2	10.5%
J	22	5	22.7%
K	21	4	19.0%
L	19	2	10.5%
M	23	0	0.0%
N	24	4	16.7%
O	18	2	11.1%
P^a^	18	0	0.0%
Q^a^	17	0	0.0%
R^a^	18	0	0.0%
S^a^	21	0	0.0%
**Total**	374	39	10.4%
**Total considering clubs that participated in both LNF 2013 and 2016**	300	39	13.0%

Source: Adapted from Marques and Marchi Jr. (2021).

Clubs were not identified for ethical reasons.

a Clubs that did not participate in LNF 2016.

**Table 2:** Players' working period in the same clubs between LNF 2016 and LNF 2019.

**Clubs of LNF 2016**	**Players in LNF 2016**	**Players that worked at the same club in both LNF 2016 and LNF 2019**	**Percentage of players that worked at the same club in both LNF 2016 and 2019**
A	21	3	14.3%
B	17	3	17.6%
C	20	3	15.0%
D	19	1	5.3%
E	24	2	8.3%
F^a^	16	0	0.0%
G	22	2	9.1%
H	19	2	10.5%
I^a^	18	0	0.0%
J	19	4	21.0%
K	22	1	4.5%
L^a^	21	0	0.0%
M^a^	24	0	0.0%
N	24	4	16.7%
O	21	2	9.5%
T^a^	17	0	0.0%
U	24	2	8.3%
V	24	2	8.3%
W	20	4	20.0%
**Total**	392	35	8.9%
**Total considering clubs that participated in both LNF 2016 and 2019**	296	35	11.8%

Source: Adapted from Marques and Marchi Jr. (2021).

Clubs were not identified for ethical reasons.

a Clubs that did not participate in LNF 2019.

### 3.3 Statistical analysis

We applied the Chi-square test to compare the frequency of the LNF athletes born in each region of Brazil with the population of Brazilian men between 0 and 14 years old in the same locations, with a significance level of α < 0.05.

To compare the data of athletes' birthplaces in different regions of Brazil among the LNF 2013, LNF 2016, LNF 2019, and LNF 2022, we used descriptive statistics (absolute numbers and percentage distribution). The same was done to analyze the athletes' working period in the same club. Data were analyzed using the software Microsoft Excel 2019 and SPSS (*Statistical Package for Social Science*) for Windows^®^ version 20.0.

## 4 Results

We divided the results section into two subsections: (a) Players' birthplaces and migratory flow; and (b) Players' working period in the same LNF club.

### 4.1 Players' birthplaces and migratory flow

Table 3 shows, in percentage and absolute numbers, the distribution of players' birthplaces in the five Brazilian regions (North, Northeast, Centre-West, Southeast, and South), considering the LNF 2013, LNF 2016, LNF 2019, and LNF 2022 seasons.

**Table 3:** Absolute numbers and percentage of LNF players' birthplaces by Brazilian regions.

**Regions**	***LNF2013***	***LNF2016***	***LNF2019***	***LNF2022***
North	3 (0.9%)	1 (0.4%)	0 (0%)	4 (1%)
Northeast	35 (9.3%)	22 (9.9%)	40 (11.7%)	48 (12%)
Centre-West	5 (1.3%)	1 (0.4%)	1 (0.3%)	9 (2.2%)
Southeast	166 (44.1%)	93 (41.8%)	146 (42.5%)	172 (42.9%)
South	167 (44.4%)	106 (47.5%)	156 (45.5%)	168 (41.9%)
**Total**	376 (100%)	223 (100%)	343 (100%)	401 (100%)

Source: The authors.

The South and Southeast regions are together the birthplaces of a range between 84.8% (LNF, 2022) and 89.3% (LNF, 2016) LNF players, with no < 41.1% in each of them in the same year. Consequently, the North, Northeast, and Centre-West regions are together the birthplace of a range between 10.7% (LNF, 2016) and 15.2% (LNF, 2022) of LNF players; the North region in LNF 2019 was the birthplace of no athlete. According to data, there is a predominance of players' birthplaces in the South and Southeast regions, very similar to the distribution of LNF clubs' locations, as shown in Table 4.

**Table 4:** Absolute numbers and percentage of LNF clubs' locations in Brazilian regions.

**Regions**	***LNF2013***	***LNF2016***	***LNF2019***	***LNF2022***
North	0 (0.0%)	0 (0.0%)	0 (0.0%)	0 (0.0%)
Northeast	0 (0.0%)	0 (0.0%)	0 (0.0%)	0 (0.0%)
Centre-West	0 (0.0%)	0 (0.0%)	0 (0.0%)	0 (0.0%)
Southeast	7 (36.8%)	5 (26.3%)	5 (26.3%)	7 (31.8%)
South	12 (63.2%)	14 (73.7%)	14 (73.7%)	15 (68.2%)
**Total**	19 (100%)	19 (100%)	19 (100%)	22 (100%)

Source: The authors.

Table 5 presents the results of the comparison of birthplaces by regions between the LNF athletes and the Brazilian men population between 0 and 14 years old in the years 1991 and 2000.

**Table 5:** Chi-square test results and frequency distribution of birthplaces by regions between the LNF athletes and the Brazilian men population between 0 and 14 years old in 1991 and 2000.

**Regions**	**LNF 2013**	**Segmented population 1991**	**LNF 2016**	**Segmented population 1991**	**LNF 2019**	**Segmented population 2000**	**LNF 2022**	**Segmented population 2000**
North	0.9%	8.4%	0.4%	8.5%	0%	7.6%	1%	7.6%
Northeast	9.3%	32.6%	9.9%	32.6%	11.7%	28.2%	12%	28.2%
Centre-West	1.3%	6.5%	0.4%	6.5%	0.3%	6.8%	2.2%	6.8%
Southeast	44.1%	38.6%	41.8%	38.5%	42.5%	42.6%	42.9%	42.6%
South	44.4%	13.9%	47.5%	13.9%	45.5%	14.8%	41.9%	14.8%
**Total**	100%	100%	100%	100%	100%	100%	100%	100%
*X* ^2^	358.5		246.5		59.1		271.0	
*p*	<0.001^*^		<0.001^*^		<0.001^*^		<0.001^*^	

* α < 0.05.

Source: The authors.

Data show that the LNF players' birthplaces are distributed in Brazilian regions differently from the segmented population (Brazilian men between 0 and 14 years old in the years 1991 and 2000). These data demonstrate that LNF players are a particular population, being influenced by a specific social context (Brazilian men's professional futsal).

### 4.2 Players' working period in the same LNF club

This subsection presents descriptive statistics (absolute numbers and percentual values) about the intra-national migration of LNF athletes. We compared how many players worked at the same club during the interval time of 3 years, and analyzed the migratory flow of athletes within this scenario. Data related to the analysis involving the LNF 2013, 2016, and 2019 (Tables 1, 2) were obtained from the study of Marques and Marchi Jr. (2021) and were exposed in the methods section of the present article.

Following, we present Table 6, which was produced by us to show data from the comparison between the LNF 2019 and LNF 2022, as a continuation of the analysis on the comparisons between the LNF 2013, 2016, and 2019 (Tables 1, 2).

**Table 6:** Players' working period in the same clubs between LNF 2019 and LNF 2022.

**Clubs of LNF 2019 and 2022**	**Players in LNF 2019**	**Players who worked at the same club in both LNF 2019 and LNF 2022**	**Percentage of players who worked at the same club in both LNF 2019 and 2022 (%)**
A	18	3	16.6
B^a^	17	0	0.0
C	20	1	5.0
D	19	3	15.7
E	22	3	13.6
G	18	1	5.5
H^a^	17	0	0.0
J	18	6	33.3
K	21	2	9.5
L	21	1	4.7
N	21	2	9.5
O	18	3	16.6
V	20	2	10.0
W	16	2	12.5
X	15	5	33.3
Y	22	5	22.7
Z	15	1	6.6
AA	17	0	0.0
AB	20	0	0.0
AC	31	7	22.5
**Total**	386	47	12.2
**Total considering clubs that participated in both LNF 2019 and 2022**	352	47	13.3

Source: The authors.

Note: Clubs were not identified for ethical reasons.

a Clubs that did not participate in LNF 2022.

Table 1 shows that 13% of the LNF 2013 athletes worked at the same club in LNF 2016. Table 2 presents that 11.8% of the LNF 2016 players were linked to the same club in LNF 2019. Table 6 indicates that 13.3% of the LNF 2019 players continued playing for the same club in LNF 2022. All data are similar, with around 11.8% and 13.3% of the LNF players having worked at the same club for 3 years. It means that LNF had a regular players' permanence rate throughout 2013 and 2022, and that there exists labor instability, with a range between 86.7% and 88.2% of the LNF players moving to another club/city with <3 years of stay in the same workplace.

## 5 Discussion

The LNF athletes' migratory flow was regular between 2013 and 2022. Data showed that athletes who were born in North, Northeast, and Centre-West regions had less participation in LNF clubs, and because of this, the league clubs don't take advantage of many talented players from these places. Considering that the LNF represents the elite of Brazilian men's futsal, it is a contradiction that, in several seasons, there were no elite clubs in some regions, as well as no elite job opportunities in these places, making this league not a national, but a regional scope championship.

Furthermore, this contradiction is also explicit because other men's elite sport leagues in Brazil present different scenarios of clubs' locations. Possible examples are the men's volleyball, basketball, and football leagues. All of them count on clubs from Northeast and/or Centre-West regions in the 2025 league editions, while LNF has not counted on clubs from these places since the 2019 edition.

The Brazilian Men's Volleyball Superleague counts on 2 clubs from the Centre-West region (Superliga, 2025). The *Novo Basquete Brasil*—NBB (Brazilian men's national basketball championship) counts on 1 club from the Centre-West and 2 from the Northeast regions (LNB, 2025). The Brazilian Men's Football Championship counts on 4 clubs from the Northeast region (CBF, 2025). In every year since 2008, both the Basketball and Football men's leagues have had at least one club from the North, Northeast, or Centre-West regions (CBF, 2025; LNB, 2025).

Another possible comparison with the LNF's scenario can be made with the Argentinian Men's elite Futsal league. This country presents several similar socioeconomic characteristics in comparison to Brazil. It has a large territory, a large border with Brazil, and is also a Global South country with a considerable social inequality context (Peres, 2024). However, the Argentinian National Futsal League (LNFA) has more egalitarian clubs spreading across the country's regions compared to Brazil. This championship has a regional preliminary phase (in which clubs from all provinces of the country participate). The best-placed clubs play the inter-regional phase. The winner of each group qualifies for the national final phase. The latter counts on 8 clubs, with the four winners of the inter-regional phase, added to four clubs from the “metropolitan phase”, composed by the champion of the LNFA in the previous season, the champion of the “Copa Argentina”, the champion of the “Primera División”, and the champion of the “Copa Oro of the Primera División” (AFA, 2024). The main men's elite futsal league in Argentina counts on clubs from all regions of the country, differently from the LNF in Brazil, offering opportunities to join LNFA clubs for players from different places.

We cannot take the LNF scenario as a standard that would be repeated in all sports in Brazil, but it is possible to affirm that the Brazilian men's elite futsal context promotes a social reproduction of the opportunities to develop a professional elite futsal career. It confirms our second hypothesis. The LNF works as a symbol of social reproduction in Brazil. There is a considerable difference in joining LNF clubs among players that were born in socioeconomic wealthier (mainly Southeast and South) and poorer regions of the country (mainly North and Northeast), being the birthplace, in a Bourdieusian perspective, a type of symbolic capital that provides certain symbolic power to privileged athletes, what confirms our first hypothesis.

Comparing the LNF athletes' migratory flow data with the intra-national (domestic) mobility of the Brazilian general worker population, there are differences among these groups that reinforce the social reproduction of unequal labor opportunities in the context of men's elite futsal in Brazil. Considering the migratory flow of Brazilian general migrant workers between the years 2010 and 2015, the North, Northeast, and Southeast regions had more emigrants than immigrants, with a high migratory flow between the latter two (Dota and Queiroz, 2019), which is very divergent from the context presented by the data in our present study.

Within this context, it is possible also to consider the Centre-West region as the third major Brazilian general migrant workers' destiny (20.47%), and the South region as the fourth one (14.29%) (Dota and Queiroz, 2019), which is considerably different from the Brazilian professional men's elite futsal scenario in all the LNF 2013, LNF 2016, LNF 2019, and LNF 2022 editions, with no migratory flow of elite athletes to the Centre-West region (no clubs were located there), and the higher concentration of clubs in the South region. This Brazilian futsal context is also divergent from the historically high Brazilian general workers' migratory flow from the Northeast to other regions of the country, mainly the Southeast and Centre-West (Baeninger, 2012), which also does not occur in the professional men's elite futsal context. In other words, despite the high concentration of LNF clubs (that can be interpreted as labor position opportunities) in the Southern regions of the country, there is not a high migratory flow of athletes from other places, as these job positions are mainly occupied by players that were born in South and Southeast, in a very regionalized athletes' mobility. Again, the birthplace seems like a symbolic capital that can provide better opportunities to develop a professional sport career.

In a different landscape, comparing the context of this present study with the scenario of athletes' transnational migration from a worldwide perspective, the migratory flow mainly happens from Global South countries, which drains sport talents to the wealthier economic centers—Global North countries -, as exemplified by data related to worldwide men football migratory flow of athletes in the studies of Poli (2010) and Poli and Ravenel (2018).

However, our data show that in the Brazilian men's elite futsal context, the intra-national migratory flow works differently from the worldwide scenario, but still reproduces socioeconomic inequality. In this case, the South and Southeast regions present the major concentration of LNF clubs in Brazil but do not receive many athletes from other regions of the country, privileging the offer of career development opportunities to players born in the Southern states. It is a perverse social reproduction scenario because there is no equitable offer of opportunities to develop a professional elite futsal career in the main men's national league (LNF) to those players born in North, Northeast, and Centre-West regions, segregating many of them from this sport market.

Our data suggest that those athletes who were born in North, Northeast, and Centre-West regions seemed to have difficulties investing in an elite professional futsal as a possibility of an elite labor career, which can also impact their job decision-making, in other words, their habitus (Bourdieu, 1998). The lack of opportunities for these athletes to develop a professional career can limit their processes of habitus transformation during migration, making it harder to compete and train among the elite players of the country in LNF. In other words, their possibilities of becoming better and more competitive players are limited.

This is the effect of the social reproduction (Bourdieu, 1973; Bourdieu and Passeron, 1990). The individuals who had better opportunities to enhance their athletic skills in their birthplace region (those born in the Brazilian Southern regions) also had favorable conditions to develop professional futsal careers. On the other hand, the players who were born in regions without LNF clubs also had fewer opportunities to join these sports institutions. This scenario represents a tendency of the privileged birthplace to influence the possibilities of capitals' conversion (Bourdieu, 1986). For example, the symbolic capital (born in the South or Southeast regions) is converted into economic capital (the salary in an elite LNF club).

Even with the tendency to reproduce their social initial conditions and the instability of a professional career landscape in Brazilian futsal, it is known that when an athlete moves from a region without LNF club to Southeast or South, it is possible to transform his habitus and to accumulate capitals, as economic (being a consequence of the sport job contract and sponsorships), social (networks), cultural (knowledge about the game and sociocultural issues in the receiving place) and symbolic (athletic performance) (Križaj et al., 2016; Smith et al., 2019; Ungruhe and Agergaard, 2020b; Painter and Price, 2021; Marques et al., 2022). So, this information is important and useful for LNF organization systems, to create opportunities for the players from the North, Northeast, and Centre-West Brazilian regions to enhance their athletic skills, join an LNF club, develop their professional futsal career, and influencing other dimensions of their personal life (as economic, for example).

Thus, the players who were born in the South and Southeast regions had better opportunities to train and compete among elite players during a longer period of their careers. In other words, they can accumulate more capitals and improve their skills by sharing experiences with other elite players (Hancock et al., 2013; Marques, 2019). Considering that migration can be interpreted as an opportunity to develop athletic performance (Barker-Ruchti and Schubring, 2016; Lago-Peñas et al., 2019; Marques et al., 2022), chances of a futsal career development were more offered to South/Southeast-born athletes (within an inter-regional mobility scenario) unequally compared to those from the North, Northeast, and Centre-West regions (who cannot try the same migration experiences).

There is a symbolic power (Bourdieu, 1989) imposed by the dominant agents of this field (futsal clubs), which decreased the opportunities for developing an elite sport career for the athletes who were born in the North, Northeast, and Centre-West regions. The LNF manager board could reflect on how to promote a more equitable distribution of capitals among Brazilian futsal players from different regions of the country, trying to decrease the effects of the reproduction of social inequalities in this context. It can be interesting to LNF because migrant athletes (in this case from the North, Northeast, and Centre-West regions) can influence the development of new talents and the level of competition in the receiving places (Elliott and Weedon, 2011; Almeida and Rubio, 2018; Painter and Price, 2021).

Furthermore, at the same time that only a few athletes were born in the North, Northeast, and Centre-West, a minority of LNF players continued joining the same club for at least 3 years. It means that there is a high men futsal players' migratory flow in Brazil, producing an unstable labor conditions context for the majority of athletes. So, this scenario let us consider that Brazilian men elite futsal athletes' careers are unstable and threatening in two ways: for athletes who were born in North, Northeast, and Centre-West regions there is a lack of opportunities to play in an LNF club; for athletes from all regions, the job agreements/contracts with the clubs seemed to work in short-term, continuing the tendency presented by Marques and Marchi Jr. (2021).

This picture of the men's elite futsal market in Brazil can also affect athletes' family relocation and socialization, which influences children's education, friendship ties, and personal/professional goals/careers of players' partners (Roderick, 2014; Van der Meij and Darby, 2017; Marques and Marchi Jr., 2021). Short-term contracts restrict the accumulation of cultural and social capitals, because players don't have enough time to establish themselves in a new city/network (Ungruhe and Agergaard, 2020a; Marques and Marchi Jr., 2021). Thus, the unpredictability of their career continuity diminishes the benefits that came from these capitals' conversions (Bourdieu, 1986) and also constraints, for example, dual-career possibilities (Ungruhe and Agergaard, 2020b; Coelho et al., 2021), which influence the reinsertion of these individuals into another profession during their post-career (Martins et al., 2021; de Souza et al., 2022). Considering that migration is related to social agents' habitus transformation from migrant to local (Bourdieu and Wacquant, 2000; Bourdieu, 2004; Bourdieu and Sayad, 2004), the lack of time and opportunities to become a member of a well-established network is a barrier to the social integration and acculturation of the migrant (Friberg, 2012; Ungruhe and Agergaard, 2020a; Shekriladze and Javakhishvili, 2024).

These unstable labor conditions are generally caused by short and/or precarious job agreements clubs offer to players, as a consequence of the deficient legal, marketing, and labor relations within sport field (Besnier et al., 2018). As exposed before, Brazilian men's elite futsal athletes (mainly born in the South and Southeast regions) move to different cities several times during their careers, and it seems not to be only an intentional choice. It is necessary to occupy better job positions in the elite sports field (Roderick, 2014; Agergaard et al., 2023). This unstable scenario is based on symbolic violence (Bourdieu, 1989) from clubs over players, creating forced labor migration in several cases, mainly because of the short-term or the breaking of job agreements (Marques and Marchi Jr., 2021). Following this, the constant mobility and high migratory flow of athletes could be interpreted as a response to the lack of equitable opportunities across the country's different regions and, consequently, to the fragile labor conditions in which men futsal athletes are involved in Brazil.

This complex context presents a paradoxical scenario. While there exists a tendency of social reproduction of job opportunities for those privileged Southern-born players, at the same time, these same athletes are exposed to an unstable labor condition that forces constant migration among cities within the Southeast and South regions, lasting short periods, in the search for better working conditions.

According to Bourdieu (2001), the neoliberal system has made precarious the most diverse labor sectors. The public sector, through temporary and/or interim positions; the private sector, through ideologies imposed by large corporations, cultural broadcasting companies, educational institutions, and the media. However, this precariousness seems to occur more clearly for agents who participate in this system as employees, in the case of this study, the Brazilian men futsal players. For them, employment as a professional athlete several times is considered a privilege. However, it is a fragile job relationship, due to the instability and the high competition in the labor world. In this context, men futsal players in LNF must constantly migrate among clubs and cities, within a horizon of inequality and instability.

## 6 Concluding thoughts

The aims of this study were: (a) to analyze how men professional futsal players' migratory flow occurs in Brazil, considering and relating the athletes' birthplace and the clubs' location regions; (b) to investigate players' working period in the same club and the tendencies of instability/stability in job positions; (c) to analyze relations between the socioeconomic inequality in different Brazilian regions and the athletes' migratory flow.

Our results showed that the majority of the Brazilian men elite futsal athletes were born in the South and Southeast regions of Brazil and highlighted the regularity in this scenario over the last decade, considering the LNF 2013, LNF 2016, LNF 2019, and LNF 2022. Then, we concluded that the LNF is regionalized in the South and Southeast, with a monopoly of the clubs concentrated in these places and a small migratory flow from different regions. There is a lack of opportunities for migration and participation in the Brazilian men's elite futsal context for those players born in the North, Northeast, and Centre-West regions, even though futsal is a very common sport practice and part of the popular culture in all Brazilian regions.

Furthermore, we found a high athletes' migratory flow among LNF clubs and Southern cities. While there is a small mobility from other regions to the South/Southeast, at the same time, there are also intense athletes' moving within and between Southern regions.

We can still consider that the elite labor career in men's Brazilian futsal is unstable, with a fragile connection between athletes and clubs, which allows players to migrate to keep their athletic habitus and to survive in the elite futsal market, making it possible to continue developing their professional elite careers. This context also reveals the fragility of the LNF organization system in two directions: the league does not take advantage of several sport talents that were born away from the South/Southeast regions; the league restricts career opportunities and, in doing it, imposes limitations to habitus transformation and skills improvement to those players that do not have the opportunity to develop a futsal elite career in a LNF club.

Within this scenario, we consider it relevant that LNF managers could plan a league structure transformation to provide better job stability for athletes in their clubs. In the same sense, we suggest that LNF managers could promote wider exchange/mobility opportunities for athletes born away from the South/Southeast regions to participate in the league. It could be done with advances in two scenarios: to stimulate the participation of clubs from the North, Northeast, and Centre-West regions (what has already happened in some editions of LNF, but not in a consistent and long-term way, mainly because fragile clubs' financial conditions/resources); and to hire more athletes that were born in these places.

The LNF clubs are commercial franchises that operate as companies, with sponsors linked to different economic sectors. They are established mostly in-country cities, presenting strong identification with the local population. The symbolic violence practiced by the LNF creates a scenario of social reproduction. Those clubs with greater prestige, located in the wealthier regions, tend to remain in better positions, while those new in the field tend to have difficulties in accessing the league's elite. The recent struggles between LNF and CBFS for the symbolic and economic power in the Brazilian futsal sports market do not allow us to say which actions will promote more egalitarian and less precarious labor opportunities for athletes, especially those born in the North, Northeast, and Central-West regions. However, this is a topic that will demand investigation soon, mainly because of the creation of the new Brazilian Futsal Championship. It is a symbol that the LNF must reflect on changing its structure in the coming years.

The main limitation of this quantitative study was the focus on a macro analysis of the LNF athletes' migration landscape. An in-depth qualitative analysis of players' perspectives in this context, as well as other social agents involved in the futsal social field, such as coaches, managers, and players' families, can be fruitful topics for future studies to understand these agents' perspectives about sport migration/labor conditions, mainly after a significant period inserted in this new landscape (LNF and Brazilian Futsal Championship).

Other possibilities for forthcoming research are to reproduce this study in the Brazilian women's futsal scenario and to analyze men's futsal context in the North, Northeast, and Centre-West regions. A better understanding of the opportunities for the development of futsal at these places, and an in-depth diagnosis of how they are organized to develop sport careers, can be relevant topics to managers and several agents within this field. More than this, it can be a first step to extend the analysis from this present study to other Brazilian sport leagues, offering a wider understanding of sport field in Brazil. The same is true for other countries' sport contexts.

## Data Availability

The raw data supporting the conclusions of this article will be made available by the authors, without undue reservation.
