# Correction: Birthplace as a capital: migratory flow, labor opportunities, and social reproduction in Brazilian men elite futsal players' careers

**DOI:** 10.3389/fsoc.2025.1675052

**Published:** 2025-08-14

**Authors:** Iuri Salim de Souza, Murilo dos Reis Morbi, Illgner Veber Garcia Alves, Christiano Streb Ricci, Renato Francisco Rodrigues Marques

**Affiliations:** ^1^University of São Paulo, Ribeirão Preto, Brazil; ^2^Marista School, Ribeirão Preto, Brazil; ^3^University of Ribeirão Preto, Ribeirão Preto, Brazil

**Keywords:** domestic move, labor mobility, sport career, Pierre Bourdieu, reflexive sociology, social inequality, social reproduction

An incorrect Funding statement was provided. The correct funder is Coordenação de Aperfeiçoamento de Pessoal de Nível Superior – Capes.

The updated funding statement is:

“The author(s) declare that financial support was received for the research and/or publication of this article. This article was supported by funding from the Coordenação de Aperfeiçoamento de Pessoal de Nível Superior – Capes (Grant Nos. 536/2021 and 1182/2022).”

The original version of this article has been updated.

